# Melanoma in Organ Transplant Recipients: Incidence, Outcomes and Management Considerations

**DOI:** 10.1155/2012/404615

**Published:** 2012-11-25

**Authors:** Faisal R. Ali, John T. Lear

**Affiliations:** The Dermatology Centre and Manchester Academic Health Science Centre, University of Manchester and Salford Royal NHS Foundation Trust, Manchester M6 8HD, UK

## Abstract

The incidence of melanoma continues to increase year on year. With better surgical techniques and medical management, greater numbers of organ transplants are being performed annually with much longer graft survival. The authors review our current understanding of the incidence of melanoma amongst organ transplant recipients, outcomes compared to the immunocompetent population, and management strategies in this burgeoning group.

## 1. Introduction

Malignant melanoma is the sixth most common UK cancer whose incidence continues to increase year on year. Whilst new therapies are being developed, advanced metastatic melanoma is invariably fatal. The number of transplant procedures performed annually continues to increase, partly owing to our ageing population. Moreover, improved surgical techniques and refined posttransplantation medical care have prolonged the longevity of organ transplant recipients (OTRs) [1] and have permitted patients to receive transplants at older ages. 

Organ transplant recipients (OTRs) are predisposed to many types of malignancies, most prominently skin cancers, owing to the considerable doses of immunosuppressive medications required [2, 3]. Nonmelanoma skin cancers (NMSCs) are most notably increased, with an increased NMSC risk of up to 250 times than that of the normal population and reversal of the ratio of basal cell carcinoma to squamous cell carcinoma [4]. As such, the National Institute of Clinical Excellence advocates annual skin surveillance should be undertaken in dedicated secondary care transplant clinics [5]. As melanoma is purported to be an immunologically mediated tumour [6] which can be treated using immunotherapy, immunosuppressive medications used by the OTR cohort could be expected to alter its natural history.

Melanoma in OTRs can arise in three principal contexts: melanoma may preexist prior to transplantation, develop following transplantation or may be derived from the organ donor. Each stratum has distinct aetiological and management considerations and will henceforth be considered separately.

## 2. Preexisting Melanoma Prior to Transplantation

### 2.1. Incidence and Outcomes

The largest study of OTRs with a history of melanoma prior to transplantation suggests no increased risk of recurrence of local or metastatic melanoma (with mean followup of 10.5 years after original melanoma diagnosis) compared to the control population [7]. This study interrogated database records of the Mayo Clinic and identified melanoma in 59 patients (61 cases) prior to transplantation. These findings supported a smaller retrospective analysis of 9 melanomas by the skin care in organ transplant patients in europe (SCOPE) [8] group with no recurrences reported with a mean of 60 month followup.

A previous interrogation of the Cincinnati Transplant Tumour Registry (CTTR) identified 31 OTRs as having a diagnosis of melanoma prior to transplantation, of whom six developed recurrent disease and died 6 to 30 months posttransplantation (32 month followup), leading the author to recommend an interval of five years following treatment prior to considering solid organ transplantation [9].

Apparent differences in reported studies may be due to paucity of histological data available. Whilst Breslow thicknesses were reported in 25% of the Mayo Clinic records and 67% of the SCOPE cohort, they were not available for any of the CTTR group which may have been of greater depth. None of these studies provide conclusive evidence for recommending an optimal interval postmelanoma prior to transplantation.

## 3. Primary Melanoma Developing Posttransplantation

### 3.1. Incidence

Amongst the OTR population, incidence of primary melanoma has been reported as between the same as up to eight times to that of the general population [10–13]. Such large variation is in part attributable to low absolute numbers of cases within each study [14]. 

The largest cohort study used data from the US Scientific Registry of Transplant Recipients (1987–2008) and included 175,732 patients with renal, cardiac, liver, and lung allografts [3]. 381 cases of melanoma were observed, suggesting an increased risk of 2.6 times than that of the general population. Similarly, a large combined Australasian registry-based prospective cohort study captured 28,855 patients with up to 42 years of followup and suggested an increased standardised incidence ratio of melanoma of 2.53 amongst the renal transplant recipient population [15]. 

Interrogation of Mayo Clinic records revealed 638 patients with posttransplantation melanoma (724 cases) with an average time of 5.5 years between first transplant and melanoma diagnosis [7].

One study has addressed the incidence of skin cancers in the paediatric posttransplant population [16]. Interestingly, skin cancers are the second most common form of malignancy after lymphoproliferative disorders in the paediatric posttransplant population. A greater proportion of these skin cancers (12%) are malignant melanoma in the paediatric versus the adult posttransplant population. These findings require validation in a larger cohort of patients.

### 3.2. Outcomes

Where melanoma occurs posttransplantation, overall and melanoma cause-specific 3-year survival is significantly worse in OTRs compared to the nontransplanted control population, with the difference most marked in cases of greater Breslow thickness. Brewer et al. reported three-year survival of 51.2% amongst patients with Breslow thickness 1.51–3.00 mm compared to expected survival of 87.4% from patients with melanoma in nontransplanted control population [7]. Subgroup analysis of cardiac transplant patients, who typically take higher doses of immunosuppressants and are at greater risk of squamous cell carcinomas [17], suggest statistically significant melanoma cause-specific mortality with Breslow thickness greater than 1.51 mm. These findings suggest that immunosuppressants are more likely to exacerbate tumours of greater Breslow thickness, affording further credence to the hypothesis that melanoma is an immunologically mediated tumour [6].

These findings were mirrored in an earlier retrospective, multicentre analysis of 91 melanomas occurring posttransplantation [8]. Compared to demographic- (age and sex) and phenotypic- (tumour thickness and ulceration status) matched controls, OTRs developing posttransplantation melanomas had similar survival outcomes for T1 and T2 tumours (less than 2 mm thickness), but significantly worse for T3 and T4 tumours (thickness greater than 2 mm). In all these cases, wide local surgical excision was undertaken. There was no statistical significance in mortality whether immunosuppression was altered or not. Posttransplantation melanoma resembles melanoma seen in the general population clinically and histologically. There appears to be no site predilection for posttransplant melanoma, unlike that for backs in males and lower limbs in females in the nontransplant population [8, 9].

### 3.3. Management Considerations

Initial treatment for posttransplant primary melanoma does not differ significantly from the nontransplant population, where early diagnosed primary melanoma can be completely surgically excised, with depth of invasion governing the excision margin and need for sentinel lymph node biopsy [18, 19].

An expert consensus survey issued by the International Transplant Skin Cancer Collaborative (ITSCC) and the Skin Care in Organ transplant Patient Europe (SCOPE) advocated reduction of immunosuppression in patients with numerous or life-threatening skin cancers [20]. The guidance attempts to balance the risk of tumour burden and risk of metastasis versus the increasing likelihood of organ rejection observed with more aggressive reduction of immunosuppression. Reduction of immunosuppression may represent dose reduction or withdrawal of one immunosuppressive agent of several being used. The types of transplanted organ (heart, liver, and kidney) were each considered separately as each imparts differing immunogenicity potential, and failure of each type of graft has different implications with respect to organ replacement therapy and clinical outcomes. Recommendations for reduction of immunosuppression for various stages of melanoma in various organ recipients are shown in Table 1, graded mild, moderate, and severe. 

In the case of renal allografts, many experts had a lower threshold for reduction of immunosuppression as dialysis was felt to offer a reasonable alternative to organ failure. For liver recipients, doses of immunosuppressive medication can be slowly reduced to lower levels, as the liver has regenerative potential following insults such as acute rejection. The corresponding threshold for reducing immunosuppression in heart transplants is considerably higher. Owing to poor renewal potential of cardiac muscle and inevitable fatality should, the graft will be rejected and subsequently fail [21]. In all cases, as morbidity and mortality of skin cancer rises, transplant physicians are more likely to accept the risk of graft compromise associated with reduction of immunosuppression [22].

In NMSC, switching from calcineurin-based immunosuppression (e.g., tacrolimus and ciclosporin) to a mammalian target of rapamycin (mTOR) inhibitor (e.g., sirolimus and everolimus) in renal transplant recipients with a previous squamous cell carcinoma is associated with prolonged tumour-free survival and significantly fewer cutaneous squamous cell carcinomas at two years [23]. Initial observations in murine models suggest that mTOR inhibitors may have an antitumoral effect against melanoma, whilst offering protection against transplanted hearts [24]. Owing to the relative rarity of melanoma, larger cohort studies will be needed to see whether such an approach would be clinically advantageous in cases of melanoma.

Modifications of immunosuppressive regimes must be made by joint consultation of the patient with oncology, dermatology, and transplant medicine teams, assessing the risk of altering the medication upon the timecourse of the tumour and implications of organ rejection in that particular individual.

## 4. Donor-Derived Melanoma

### 4.1. Incidence

Malignancy inadvertently derived from the donor organ is a rare event relative to the number of transplants occurring annually [25]. Donors are not believed to have a significantly different incidence of skin cancer to the nondonor population [26]. Metastatic melanoma following homologous transplant of a metastatic nodule was first reported in 1965 [27]. As the number of organ transplants performed annually continues to increase and OTRs are surviving for longer periods, there is an amassing body of case reports of such cases of donor-derived melanoma [28–39]. Affected recipients developed melanoma from three months to three years after transplantation [30, 38]. 

Many organ donors, later believed to have occult melanoma at the time of organ harvesting, have been initially diagnosed with a cerebrovascular accident (CVA) or primary brain tumour as a cause of death; in retrospect, this is likely attributable to metastatic melanoma [21, 25]. A previous diagnosis of melanoma had not been made in all cases. In a recent review [21] combining case series and case reports, 17 donors have provided organs to 44 recipients, of whom 35 developed melanoma, and 24 died from metastatic disease.

### 4.2. Management

Where possible, resection of the donor organ (containing melanoma) and associated metastases should be performed together with cessation or reduction of immunosuppression [25]. This may only be a viable option in renal transplant recipients, owing to lack of long-term organ replacement therapy in the case of heart, liver and lungs. Following removal of the donated organ, retransplantation may then be possible [33, 40].

### 4.3. Selection of Organ Donors

When considering organ donors, in cases of young death purported to be from CVA or primary cerebral neoplasm, the index of suspicion for melanoma should be raised, particularly in individuals with a history of multiple previously excised moles. It has been suggested that where there is a prior history of melanoma, even if fully excised and recurrencefree for several years, this should be an absolute contraindication to organ donation, owing to the possibility of latent melanoma or ultralate recurrence [41, 42] which may be as much as 32 years after initial diagnosis and excision [43]. Additionally, comprehensive examination of the skin and evaluation for lymphadenopathy and organomegaly should be performed to exclude melanoma prior to organ donation. 

## 5. Conclusions

Stratifying melanoma into three aetiological strata allows us to draw useful lessons. Patients who have thin melanomas removed prior to organ transplantation appear not be at increased risk of recurrence following transplantation. Immunosuppressive medications taken by OTRs appear to predispose to *de novo* melanoma and are associated with worse outcomes in tumours of greater Breslow thickness. Cases of donor-derived melanoma underline the need for careful donor selection and exclusion of melanoma prior to transplantation.

As increasing numbers of organ transplants are performed, future studies may better characterise outcomes of melanoma occurring in OTRs to help inform treatment strategies in the immunocompromised population, particularly with respect to reduction and alteration of immunosuppressive medications.

## Figures and Tables

**Table 1:** Recommendation for level of reduction of immunosuppression to consider in varying stages of melanoma based on expert consensus (adapted from [20]).

Stage of melanoma	Level of reduction of immunosuppression to consider		
	Type of allograft		
	Renal	Heart	Liver
Ia	Mild	None	Mild
Ib	Mild	Mild	Mild
IIa	Moderate	Mild	Moderate
IIb	Moderate	Mild	Moderate
IIc/III	Severe	Moderate	Moderate
IV	Severe	Severe	Severe
